# The application of lymphocyte*platelet and mean platelet volume/platelet ratio in influenza A infection in children

**DOI:** 10.1002/jcla.22995

**Published:** 2019-08-16

**Authors:** Yang Fei, Hongbo Zhang, Chi Zhang

**Affiliations:** ^1^ Department of Clinical Laboratory, Tongji Hospital, Tongji Medical College Huazhong University of Science and Technology Wuhan China

**Keywords:** influenza A, lymphopenia, mean platelet volume, thrombocytopenia

## Abstract

**Background:**

To explore the characteristics and regularity of complete blood count (CBC) changes among influenza A–positive child patients and to discover parameters that can help with the diagnosis and differential diagnosis.

**Methods:**

One hundred and ninety‐one influenza A–positive children, two hundred and nineteen influenza A–negative children with influenza‐like symptoms, and two hundred and forty‐seven healthy children were included in this study. They were divided into three groups: influenza A–positive patient group, influenza A–negative patient group, and control group. Reverse transcriptase polymerase chain reaction testing and Sysmex XS‐800i hematology analyzer were used to obtain influenza A and CBC results, respectively. CBC along with parameters including lymphocyte‐to‐monocyte ratio (LMR), neutrophil‐to‐lymphocyte ratio (NLR), platelet‐to‐lymphocyte ratio (PLR), mean platelet volume/platelet ratio (MPV/PLT), and lymphocyte*platelet (LYM*PLT) was calculated and recorded for each child. The differences in these parameters among different groups were tested with SPSS 15.0. The diagnostic values were also evaluated.

**Results:**

The LYM and PLT of child patients with influenza A were significantly lower than those of both influenza A–negative patients with influenza‐like symptoms and healthy controls. Among all the parameters, LYM*PLT has the largest area under the curve and the highest diagnostic value, followed by MPV/PLT. Compared with using LMR or MPV/PLT, the diagnostic value of using LYM alone was, on the contrary, higher.

**Conclusions:**

Low LYM*PLT and high MPV/PLT may indicate influenza A infection in children with influenza‐like symptoms, which can be a useful indicator for diagnosis and differentiation of influenza A infection.

**Abbreviations**
AUC: area under the curve
CBC: complete blood count
LMR: lymphocyte‐to‐monocyte ratio
LYM: lymphocyte
MON: monocyte
MPV: mean platelet volume
NEU: neutrophil
NLR: neutrophil‐to‐lymphocyte ratio
PLR: platelet‐to‐lymphocyte ratio
PLT: platelet
ROC: receiver operating characteristic
RT‐PCR: reverse transcriptase polymerase chain reaction
SPSS: Statistical Package for the Social Sciences
WBC: white blood cell count

## 1 INTRODUCTION

Influenza A is an acute respiratory disease caused by influenza A virus leading to 3 to 5 million severe illness cases and more than 300,000 deaths during epidemics in both adults and children.^1^ The initial symptoms of influenza A are similar to those of common seasonal colds with cough, sore throat, fever, headache, and body pain. Although major influenza A infections were self‐limiting, some patients are progressing rapidly with body temperature of more than 39℃, which can develop into severe pneumonia.^2^ Additionally, severe and critical patients may suffer from decreased oxygenation and respiratory distress, which makes the mortality risk of influenza A infection greater than common seasonal colds. As there are effective treatments for influenza A and the timeliness of them is of great importance,^3^ rapidly distinguishing influenza A–positive patients from common cold patients allows for prompt antiviral therapy and judicious use of antibiotics, which lead to the reduction in transmission, morbidity, and hospitalization time.^4^ However, unlike severe influenza A, which has characteristic findings, mild or moderate influenza A is clinically indistinguishable from other influenza‐like illnesses.^5^ Early and rapid diagnosis of influenza A is challenging.^6^

Complete blood count (CBC) is one of the most routine laboratory tests being examined in patients with cold symptoms, which can be carried out in hospitals of different grades and conditions. If influenza A infection can be rapidly predicted or screened out by CBC or related results, the patients will be treated more timely with less risk of complications. Compared with adults, children have lower immunity with more dense population in active areas; thus, the risk of influenza A virus infection is higher and they are more likely to have serious complications or even death.^3^ Additionally, the reference intervals of CBC for adults and children are different. Lymphopenia has been reported in adults and children with pandemic influenza A infection in several studies.^7^, ^8^, ^9^ Lymphocyte‐to‐monocyte ratio (LMR) as a predictor of adult influenza A virus has also been reported before.^10^, ^11^ Is there diagnostic value of LMR in influenza A infection among children? Are there any other hematological parameters that can help with the diagnosis and differential diagnosis?

A retrospective study of influenza A and CBC results of patients who presented to Tongji Hospital, Tongji Medical College, Huazhong University of Science and Technology, with symptoms of influenza‐like illness during September 1, 2018, to April 30, 2019, was designed to explore the characteristics and regularity of CBC changes among child patients.

## 2 METHODS AND MATERIALS

### 2.1 Study design

Patients less than six years old with available influenza A and CBC results were analyzed in the study through reviewing cases between September 1, 2018, and April 30, 2019, in Tongji Hospital, Tongji Medical College, Huazhong University of Science and Technology, China. Inclusion criteria were fever, respiratory tract infection, cough, sore throat, headache, body pain, and pneumonia. Patients with liver disease, nephropathy, urinary tract infection, cardiovascular diseases, anemia, hematopathy, cancer, and sepsis were excluded. Reverse transcriptase polymerase chain reaction (RT‐PCR) testing and Sysmex XS‐800i hematology analyzer were used to obtain influenza A and CBC results, respectively. Fully automatic nucleic acid extractor and its associated reagents (TIANLONG NP968; Xi'an Tianlong Science and Technology Co. Ltd.) were used to extract nucleic acid from nasopharyngeal swab sent for examination. The extracted nucleic acid was then detected by fluorescent RT‐PCR with the use of Influenza A & B Virus Nucleic Acid Assay Kit (Liferiver, Shanghai Zhijiang Biotechnology Co. Ltd.) on a real‐time fluorescence quantitative PCR instrument (TIANLONG TL988‐IV; Xi'an Tianlong Science and Technology Co. Ltd). All reagent preparation, reaction conditions, and determination of results are strictly referred to related kit instructions. A total of 410 patients were included. They were divided into two groups according to influenza A results: influenza A–positive patient group (191) and influenza A–negative patient group (219). Simultaneously, two hundred and forty‐seven child cases were selected from healthy physical examination population as the control group. There was no significant difference in gender or age among the three groups. The study protocol was approved by the Tongji Hospital Ethics Committee for Research in Health (TJ‐IRB20192421).

The first‐visit CBC results including white blood cell count (WBC), neutrophil (NEU), lymphocyte (LYM), monocyte (MON), platelet (PLT), and mean platelet volume (MPV) along with influenza A results of each patient were recorded. Additionally, another five hematological parameters were calculated, which were neutrophil‐to‐lymphocyte ratio (NLR), platelet‐to‐lymphocyte ratio (PLR), LMR, MPV/PLT (MPV divided by PLT), and LYM*PLT (LYM multiplied by PLT). For details, see Table 1.

**Table 1:** Results of age, gender, complete blood count, and related hematological parameters of the influenza A–positive patient, influenza A–negative patient, and control groups

Parameters	Influenza A–positive patient group	Influenza A–negative patient group	Control group	*χ* ^2^/F	*P*‐value
N	191	219	247		
Age (d)	730.0(395.0‐1275.0)	760.0(365.0‐1245.0)	766.5(450.0‐1314.0)	0.018	.970
Boys (%)	115(60.2%)	130(59.4%)	148(59.9%)	0.032	.984
WBC (10^9^/L)	6.60(4.52‐9.00)	8.66(6.31‐12.69)^a^	8.47(7.09‐9.99)^a^	48.680	<.0001
NEU (10^9^/L)	2.89(1.44‐4.89)	3.32(1.99‐5.50)^a^	2.84(1.84‐3.92)^b^	13.360	.001
LYM (10^9^/L)	2.50(1.64‐3.95)	3.64(2.37‐5.59)^a^	4.12(3.31‐5.45)^ab^	81.570	<.0001
MON (10^9^/L)	0.57(0.33‐0.90)	0.67(0.48‐1.00)^a^	0.60(0.50‐0.81)	9.415	.009
PLT (10^9^/L)	250.00(181.00‐328.00)	317.00(239.00‐401.00)^a^	336.00(288.00‐407.00)^ab^	64.940	<.0001
MPV (fl)	10.20(9.60‐10.80)	9.90(9.30‐10.50)^a^	10.00(9.20‐10.70)^a^	13.650	.001
NLR	1.05(0.51‐2.26)	0.82(0.46‐1.94)	0.72(0.36‐1.10)^ab^	29.950	<.0001
PLR	98.38(66.10‐155.80)	82.51(56.34‐130.50)^a^	83.88(62.36‐103.50)^a^	15.000	.001
LMR	4.58(2.74‐7.43)	5.85(3.35‐8.49)^a^	6.55(5.08‐8.56)^ab^	35.950	<.0001
MPV/PLT	0.040(0.030‐0.060)	0.031(0.024‐0.043)^a^	0.029(0.024‐0.036)^ab^	65.410	<.0001
LYM*PLT	585.60(316.2‐1134)	1228.00(576.0‐1888.0)^a^	1429.00(967.1‐2000.0)^ab^	105.300	<.0001

Note

One‐way ANOVA was used for normally distributed data, the Kruskal‐Wallis H test was used for non‐normally distributed data, and chi‐square test was used for the comparison of rates.

Abbreviations: LMR, lymphocyte‐to‐monocyte ratio; LYM, lymphocyte; MON, monocyte; MPV, mean platelet volume; NEU, neutrophil; NLR, neutrophil‐to‐lymphocyte ratio; PLR, platelet‐to‐lymphocyte ratio; PLT, platelet; WBC, white blood cell count.

a Compared with the influenza A–positive patient group, *P* < .05.

b Compared with the influenza A–negative patient group, *P* < .05.

### 2.2 Statistical analyses

Statistical Package for the Social Sciences (SPSS) version 15.0 (SPSS Inc) was used for statistical analysis with *P*‐value < .05 considered as statistically significant. Continuous variables are given as mean ± standard deviation or median (interquartile rang), and categorical variables are given as percentages. One‐way ANOVA (for normally distributed data) or Kruskal‐Wallis *H* test (for non‐normally distributed data) was used for continuous variables, and chi‐square test was used for the comparison of rates. The sensitivity, specificity, positive predictive value, negative predictive value, and area under the curve (AUC) of LYM, PLT, MPV, LMR, MPV/PLT, and LYM*PLT were calculated using the receiver operating characteristic (ROC) curve. The diagnostic values of these parameters in influenza A infection were also evaluated.

## 3 RESULTS

### 3.1 Results of CBC and related hematological parameters in different patient groups

**Figure 1:**
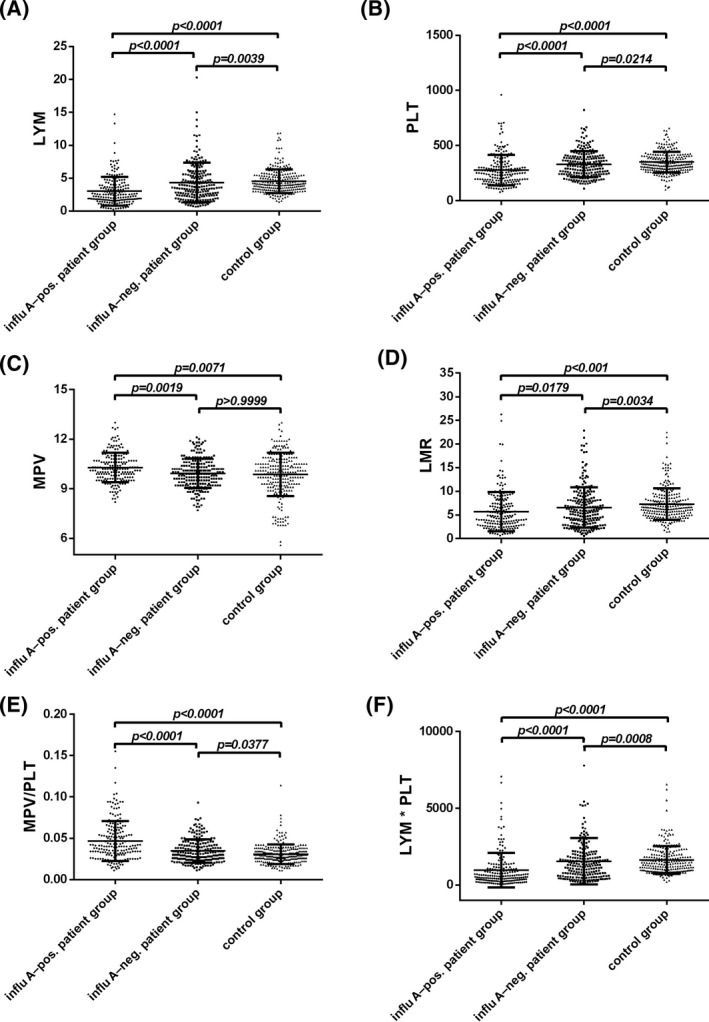
A, Distributions of lymphocyte (LYM); B, platelet (PLT); C, mean platelet volume (MPV); D, lymphocyte‐to‐monocyte ratio (LMR); E, MPV/PLT; and F, LYM*PLT in the influenza A–positive patient group, the influenza A–negative patient group, and the control group

The age, gender, and CBC results of the influenza A–positive patient, influenza A–negative patient, and control groups are shown in Table 1. There were no significant differences in age or gender among the three groups. Compared with the control group, LYM, PLT, LMR, and LYM*PLT in the influenza A–positive and influenza A–negative patient groups were significantly lower, while NLR and MPV/PLT were significantly higher. In addition, compared with patients in the influenza A–negative group, the patients in the positive group had significantly lower WBC, NEU, LYM, MON, PLT, LMR, and LYM*PLT values and significantly higher MPV, PLR, and MPV/PLT values. The distribution of LYM (median: pos. 2.50, neg. 3.64, ctrl. 4.12), PLT (median: pos. 250.00, neg. 317.00, ctrl. 336.00), MPV (median: pos. 10.20, neg. 9.90, ctrl. 10.00), LMR (median: pos. 4.58, neg. 5.85, ctrl. 6.55), MPV/PLT (median: pos. 0.040, neg. 0.031, ctrl. 0.029), and LYM*PLT (median: pos. 585.60, neg. 1228.00, ctrl. 1429.00) in different groups with *P*‐values of comparisons between two groups is shown in Figure 1.

### 3.2 Diagnostic values of LMR, MPV/PLT, and LYM*PLT for distinguishing influenza A–positive patients from suspected but influenza A–negative patients or controls

**Figure 2:**
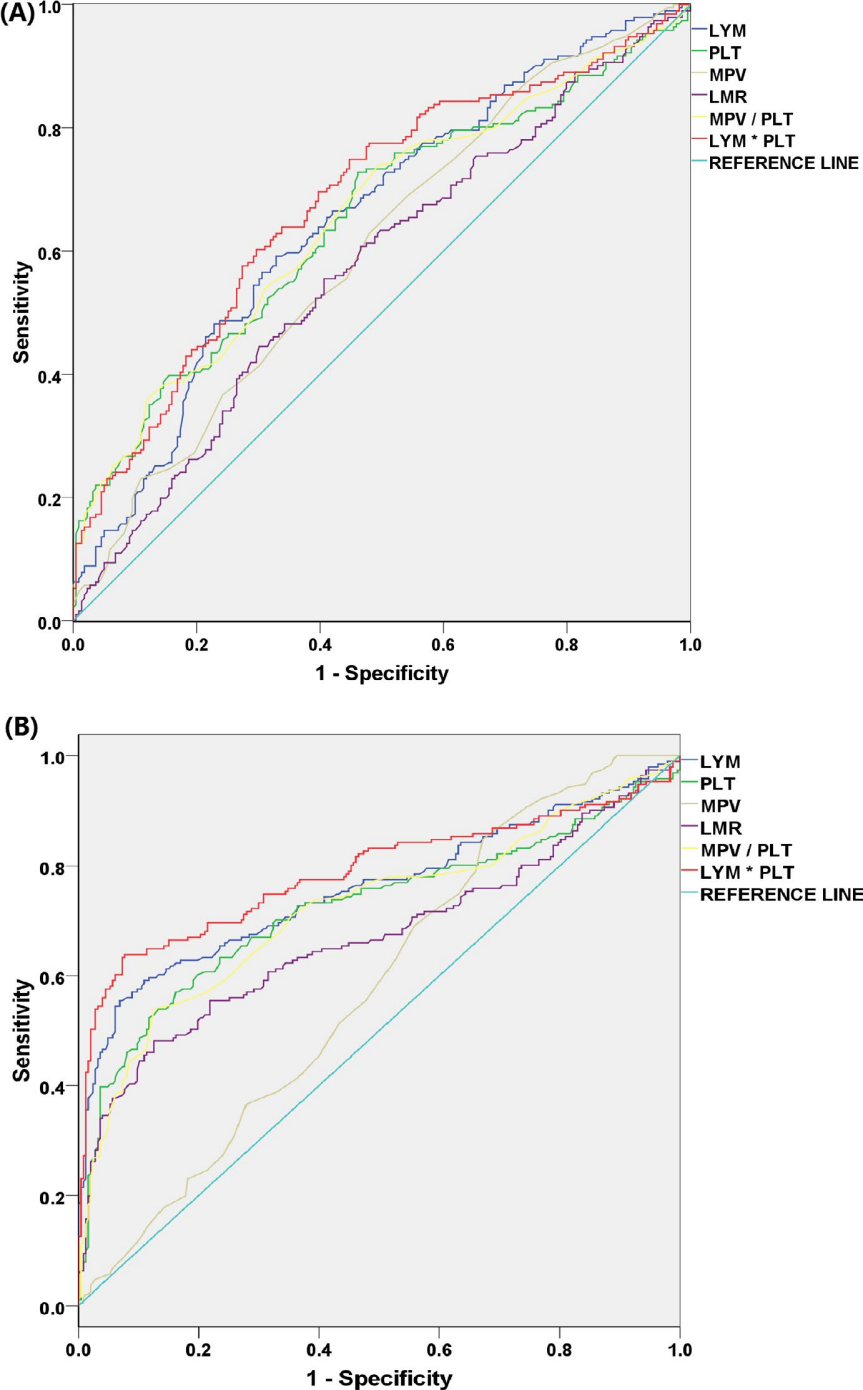
Receiver operating characteristic (ROC) curve of lymphocyte (LYM), platelet (PLT), mean platelet volume (MPV), lymphocyte‐to‐monocyte ratio (LMR), MPV/PLT, and LYM*PLT in influenza A. A, The influenza A–negative patient group was used as reference; B, the control group was used as reference

With a cutoff value of 3.98, LMR distinguished influenza A–positive patients from suspected but influenza A–negative patients with the highest sensitivity and specificity of 44.50% and 69.86%, respectively, while the sensitivity and specificity of LMR were highest at 48.17% and 87.45% in the influenza A–positive group with a cutoff value of 4.25 if controls were used as reference. MPV/PLT distinguished influenza A–positive patients from suspected but influenza A–negative patients with the highest sensitivity and specificity of 73.82% and 50.68% with a cutoff value of 0.032, while the sensitivity (53.93%) and specificity (87.45%) of MPV/PLT were highest in the influenza A–positive group with a cutoff value of 0.040 if the control group was used as reference. For LYM*PLT, the sensitivity (57.59%) and specificity (72.60%) were highest if 660.70 was used as the cutoff value with the influenza A–negative group as reference. And if controls were used as reference, the highest sensitivity and specificity of LYM*PLT were 63.87% and 92.31%, respectively, with a cutoff value of 781.55. Compared with using LYM, PLT, and MPV alone, using LYM*PLT to distinguish the influenza A–positive group from the influenza A–negative group or the control group produced a larger AUC. On the contrary, the AUC of other calculated parameters (LMR and MPV/PLT) was smaller than that of LYM when either the influenza A–negative group or the control group was used as reference. For details, see Tables 2 and 3, and Figure 2.

**Table 2:** Diagnostic performances of LYM, PLT, MPV, LMR, MPV/PLT, and LYM*PLT for distinguishing influenza A–positive patients from influenza A–negative patients with similar symptoms

Parameter	Cutoff value^a^	Sensitivity	Specificity	PPV	NPV	Accuracy	AUC (95% CI)
LYM	2.76	59.16	67.12	61.08	65.33	63.41	0.655 (0.602‐0.707)
PLT	309.50	72.77	53.88	57.92	69.41	62.68	0.650 (0.596‐0.703)
MPV	9.95	62.83	52.05	63.33	61.62	57.07	0.601 (0.547‐0.656)
LMR	3.98	44.50	69.86	56.29	56.07	58.05	0.575 (0.520‐0.631)
MPV/PLT	0.032	73.82	50.68	56.63	68.94	61.46	0.655 (0.602‐0.708)
LYM*PLT	660.70	57.59	72.60	64.71	66.25	65.61	0.682 (0.630‐0.734)

Abbreviations: AUC (95% CI), area under the receiver operating characteristic curve (95% confidence interval); LMR, lymphocyte‐to‐monocyte ratio; LYM, lymphocyte; MPV, mean platelet volume; NPV, negative predictive value; PLT, platelet; PPV, positive predictive value.

a The Youden index of receiver operating characteristic curve was the largest when this cutoff value was used.

**Table 3:** Diagnostic performances of LYM, PLT, MPV, LMR, MPV/PLT, and LYM*PLT for distinguishing influenza A–positive patients from healthy controls

Parameter	Cutoff value^a^	Sensitivity	Specificity	PPV	NPV	Accuracy	AUC (95% CI)
LYM	2.59	55.50	93.12	86.18	73.02	76.71	0.758 (0.710‐0.807)
PLT	268.50	57.07	83.81	73.15	71.63	72.15	0.723 (0.671‐0.774)
MPV	9.45	84.82	32.79	49.39	73.64	55.48	0.580 (0.527‐0.633)
LMR	4.25	48.17	87.45	74.80	68.57	70.32	0.670 (0.616‐0.724)
MPV/PLT	0.040	53.93	87.45	76.87	71.05	72.83	0.722 (0.672‐0.772)
LYM*PLT	781.55	63.87	92.31	86.52	76.77	79.91	0.788 (0.740‐0.835)

Abbreviations: LYM, lymphocyte; PLT, platelet; MPV, mean platelet volume; LMR, lymphocyte‐to‐monocyte ratio; PPV, positive predictive value; NPV, negative predictive value; AUC (95% CI), area under the receiver operating characteristic curve (95% confidence interval).

a The Youden index of receiver operating characteristic curve was the largest when this cutoff value was used.

## 4 DISCUSSION

Influenza was first clearly described by the English physician Caus in 1551 as a ‘‘sweating disease’’ characterized by fever, headache, and myalgias that killed some patients rapidly but lasted only a few days in those that survived.^12^ Over the past 300 years, there have been at least six influenza pandemics: the Spanish influenza (A H1N1) in 1918, which may have originated in the United States^13^; the Asian influenza (A H2N2) in 1957, which started from Guizhou, China^14^; the influenza A H3N2 in 1968, which originated in Hong Kong, China^15^; the Russian influenza (A H1N1) in 1977, which originated in Dandong, northeast China^16^; and the new influenza (A H1N1) in 2009, which started from Mexico and the United States.^17^, ^18^ As the subtypes are numerous and the mutations occur rapidly, influenza virus has become an important threat to public health over the world.

Normally, influenza is a relatively mild respiratory infection with high morbidity and low mortality. Although the mortality was relatively low, the total number of fatalities involved was huge, which happened more common in the very young, the very old, and the immunosuppressed.^12^ As mild‐to‐moderate influenza is clinically indistinguishable from influenza‐like illnesses caused by other respiratory viruses, laboratory tests are necessary.^5^ There are many tests that can help with the diagnosis of influenza A, such as RT‐PCR testing, virus isolation and culture, rapid detection of viral antigen, and serological antibody tests, but these tests are often unavailable especially in primary or community hospitals with poor basic conditions.^19^ The inability to rapidly diagnose or rule out influenza A presented great difficulties in infectious disease control. Under this circumstance, using routine tests to help with the diagnosis and differential diagnosis of influenza A is of great significance. We undertook this study to discover laboratory parameters to help identify influenza A infection among child patients presenting with influenza‐like symptoms while awaiting throat swab RT‐PCR or virus isolation reports. Results in our study showed that LYM*PLT could assist in the diagnosis and differential diagnosis of influenza A in children population, and had higher diagnostic value than using either LYM or PLT alone or other calculated parameters such as LMR and MPV/PLT. Besides, compared with using LMR or MPV/PLT, the diagnostic value of using LYM alone was, on the contrary, higher.

In the study, LYM of child patients with influenza A was significantly lower than that of both influenza A–negative patients with influenza‐like symptoms and healthy controls, which is consistent with the results in other studies.^9^, ^20^, ^21^ The decrease in LYM may be due to monocytes and macrophages, which regulate the expression of fALS on cell surface and release of soluble fALS to promote LYM apoptosis, as shown in the study conducted by Nichols JE et al^22^ Another study also indicated that influenza viruses might temporally destroy the human immune system's line of defense by increasing the granzyme B positive cells to kill virus‐infected LYM and MON, resulting in susceptibility to a secondary infection.^23^ Compared with controls and the influenza A–negative group, PLT in the influenza A–positive group also decreased significantly (*P* < .05). This decrease may be due to the destruction of megakaryocyte formation and the shortening of PLT cycle time, resulting in the decrease in PLT. At the same time, the virus can produce some molecules which lead to PLT adhesion and aggregation, forming circulating complexes and aggravating the decrease in PLT.^20^, ^24^

A swine influence study in India indicated that NLR < 2 along with a decrease in WBC count can be used as a screening tool in patients presenting with influenza‐like symptoms, while awaiting throat swab culture reports for confirmation.^25^ Another study in Turkey observed a significant decrease in LYM and a significant increase in MON and declared that relative lymphopenia and monocytosis may be considered as a surrogate marker of pandemic influenza A.^26^ Influenza A virus infection is initiated by hemagglutinin HA binding to sialic acid receptors in epithelial cells of respiratory tract, and the virus then replicates and spreads to other cells through endocytosis followed by the release of several cytokines. Cytokines such as CXR1/2 help convene NEU by chemotaxis and alter the movement of MON and LYM to promote their aggregation in inflammatory sites and activate specific cellular and humoral immune responses, thus increasing NEU and MON in the peripheral blood.^27^ However, in this study the increase in NEU and MON is not as obvious as expected. Meanwhile, there was no significant difference in NLR between the influenza A–positive and influenza A–negative groups, and the diagnostic value of LMR was not as good as LYM. The authors believe the reason may be that children have poorer immune function, which makes the changes happening in peripheral blood cells, ie, NEU and MON, slower and weaker.

There are many laboratory methods with different advantages and disadvantages for influenza A virus infection. The virus isolation is regarded as the reference method or “golden standard,” but it is not suitable as routine test in hospital because of its harsh, time‐consuming and laborious experimental procedure. Specific antibodies are often generated one to three weeks after virus infection, so the detection of specific antibodies is only suitable for retrospective analysis, not for early diagnosis. Rapid antigen tests with the advantages of easy operation, simple result interpretation, and fast testing speed were widely used in outpatient and emergency departments, but the high false‐negative probability has been widely reported so the results may need further confirmation of virus isolation or RT‐PCR. RT‐PCR is an important detection method for the diagnosis of influenza A.^28^, ^29^ The study performed by Cho et al 28 showed that the sensitivity and specificity of RT‐PCR were as high as 98.5% and 100%, respectively. Thus, these were chosen as diagnostic criteria in our study. However, RT‐PCR is not suitable for outpatients or emergency department patients because of the time‐consuming characteristics which may result in a significant delay in confirmation of suspected cases. Meanwhile, although the definitive test for influenza diagnosis remains RT‐PCR, during the pandemic, RT‐PCR testing was often restricted.^12^

Among all the parameters, LYM*PLT has the largest AUC and the highest diagnostic value. However, the AUC was only 0.682 with the best sensitivity and specificity of 57.59% and 72.60%, respectively, if the influenza A–negative group was used as reference. Meanwhile, using controls as reference, the AUC of LYM*PLT was 0.788 with the best sensitivity and specificity of 63.87% and 92.31%, respectively. This means the diagnostic value of hematological parameters is not good. It is worth noting that the best sensitivity and specificity of MPV/PLT are 73.82% and 50.68% if the influenza A–negative group is used as reference, which may be used in combination with LYM*PLT to improve the differential diagnostic value. However, LYM*PLT and MPV/PLT may be mainly involved in a balanced state of promoting inflammation and antiviral response in influenza virus infection. When using these parameters as diagnostic and predictive indicators, epidemiological history and comprehensive situation should be combined in order to avoid overuse and unnecessary treatment. According to the data in this study, we considered that if the LYM*PLT values were lower than 781.55 and the MPV/PLT values were higher than 0.040, the results may indicate influenza A infection in children.

There are several limitations in this study. First, relatively few cases were enrolled in this study, so large‐scale multicenter clinical studies are required to corroborate this evidence. Second, it is a retrospective study, which cannot completely resolve some confounding factors and may produce a certain degree of deviation.

## 5 CONCLUSIONS

Low LYM*PLT and high MPV/PLT may indicate influenza A infection in children with influenza‐like symptoms, which can be a useful indicator for the diagnosis and differential diagnosis of influenza A infection.

## CONFLICT OF INTEREST

The authors stated that there are no conflicts of interest regarding the publication of this article.

## ETHICAL APPROVAL

The study protocol was approved by the Tongji Hospital Ethics Committee for Research in Health (TJ‐IRB20192421).
